# The Flavonoid Isoquercitrin Precludes Initiation of Zika Virus Infection in Human Cells

**DOI:** 10.3390/ijms19041093

**Published:** 2018-04-05

**Authors:** Arnaud Gaudry, Sandra Bos, Wildriss Viranaicken, Marjolaine Roche, Pascale Krejbich-Trotot, Gilles Gadea, Philippe Desprès, Chaker El-Kalamouni

**Affiliations:** 1 Université de la Réunion, INSERM U1187, CNRS UMR 9192, IRD UMR 249, Unité Mixte Processus Infectieux en Milieu Insulaire Tropical, Plateforme Technologique CYROI, 94791 Sainte Clotilde, La Réunion, France; arnaud.gaudry@etu.unige.ch (A.G.); sandrabos.lab@gmail.com (S.B.); wildriss.viranaicken@univ-reunion.fr (W.V.); marjolaine.roche@univ-reunion.fr (M.R.); pascale.krejbich@univ-reunion.fr (P.K.-T.); gilles.gadea@inserm.fr (G.G.); 2 School of Pharmaceutical Sciences, EPGL, University of Geneva, University of Lausanne, Quai Ernest-Ansermet 30, CH-1211 Geneva 4, Switzerland

**Keywords:** Zika virus, flavivirus, antiviral activity, natural compounds, nutraceutical, polyphenols, flavonoids, isoquercitrin

## Abstract

The medical importance of Zika virus (ZIKV) was fully highlighted during the recent epidemics in South Pacific islands and Americas due to ZIKV association with severe damage to fetal brain development and neurological complications in adult patients. A worldwide research effort has been undertaken to identify effective compounds to prevent or treat ZIKV infection. Fruits and vegetables may be sources of compounds with medicinal properties. Flavonoids are one class of plant compounds that emerge as promising antiviral molecules against ZIKV. In the present study, we demonstrated that flavonoid isoquercitrin exerts antiviral activity against African historical and Asian epidemic strains of ZIKV in human hepatoma, epithelial, and neuroblastoma cell lines. Time-of-drug addition assays showed that isoquercitrin acts on ZIKV entry by preventing the internalisation of virus particles into the host cell. Our data also suggest that the glycosylated moiety of isoquercitrin might play a role in the antiviral effect of the flavonoid against ZIKV. Our results highlight the importance of isoquercitrin as a promising natural antiviral compound to prevent ZIKV infection.

## 1. Introduction

The mosquito-borne Zika virus (ZIKV), which was historically identified in Africa, has recently gained global attention due to the recent epidemics in South Pacific islands and then Americas, and its newly recognised association with Guillain–Barré syndrome and dramatic congenital malformations in infants born from infected mothers [1,2]. ZIKV can be shed in different human fluids including the semen and vaginal secretions of humans, leading to sexual transmissions. ZIKV infection could also be a cause of severe damages to sexual organs [3,4,5,6]. To date, there is no vaccination or anti-ZIKV therapy available.

The ZIKV genomic RNA encodes a single large polyprotein which is processed by host and viral proteases into three structural proteins (capsid C, membrane prM/M and envelope E), and seven nonstructural (NS) proteins: NS1, NS2A, NS2B, NS3, NS4A, NS4B, and NS5 [7,8]. The entry of ZIKV includes an initial cell attachment through the interaction between the E protein and specific cellular receptors such as tyrosine-protein kinase receptor Axl [9]. The Axl protein interacts with vitamin K-dependent protein growth-arrest-specific 6 (Gas6) for transduction of signaling pathways involved in cell proliferation. The strategy of ZIKV internalisation involves a clathrin-mediated endocytic pathway. Inside the endosomes, the E protein undergoes conformational changes, leading to ZIKV fusion with cellular membranes followed by a delivering of genomic RNA into the cytoplasm. Both viral replication and virus assembly occur at the cytosolic side of the endoplasmic reticulum membranes. Newly assembled virus particles are transported to the plasma membrane through the secretory pathway and then released into the extracellular compartment as infectious ZIKV [10].

Phylogenetic studies allowed the division of ZIKV into African and Asian genotypes [11]. Since 2007, the leading cause of major epidemics is the Asian genotype of ZIKV with tens of thousands of infection cases [12]. The characterization of contemporary epidemic ZIKV strains has advanced greatly leading to the identification of viral and cellular mechanisms that are potentially important in viral pathogenesis [9]. To combat ZIKV-related disease, it is urgent to propose safe and effective anti-ZIKV compounds not only to impair ZIKV spread, but also to mitigate ZIKV-associated morbidities. An initial effort has been made to search for antiviral treatments by testing potential ZIKV inhibitors from broad spectrum antiviral drugs [13,14,15]. It has been reported that anti-hepatitis C virus (HCV) drug sofosbuvir, adenosine analog BCX4430, and bacterially-derived compound nanchangmycin were effective against ZIKV infection [16,17,18]. Despite the development of antiviral agents from synthetic sources, a novel antiviral approach (economical, simple, and environmentally friendly) is to use natural sources—such as nutraceuticals—as preventive treatments against viral infections [19,20,21]. The family of flavonoid molecules emerged from many promising antiviral candidates [22,23,24,25,26]. Flavonoids are widely found in fruits, vegetables, nuts, seeds, flowers, tea, and wine. Intrinsically, flavonoids are the most abundant polyphenols in human diet [27]. The basic structure of flavonoids is a diphenylpropane skeleton linked by a three carbons chain that forms a closed pyran ring. Varieties of functional groups are grafted at different positions on this skeleton making the flavonoids one of the larger and diversified groups of bioactive phytochemicals [28].

Flavonoids exhibit a multitude of biological activities including antiviral activity against viruses of medical concern [29,30,31,32,33]. Recently, it has been reported that epigallocatechin gallate (EGCG), curcumin, and isoquercitrin (quercetin-3-*O*-glucoside or Q3G) are efficient against ZIKV [34,35,36]. However, very little is known about the mechanism of Q3G-mediated inhibition of ZIKV. In the present study, we demonstrated that Q3G prevents initiation of ZIKV infection in different human cells by blocking virus entry into the host cell.

## 2. Results and Discussion

### 2.1. Q3G Precludes ZIKV Infection in Human Cells of Various Tissue Origin

**Figure 1:**
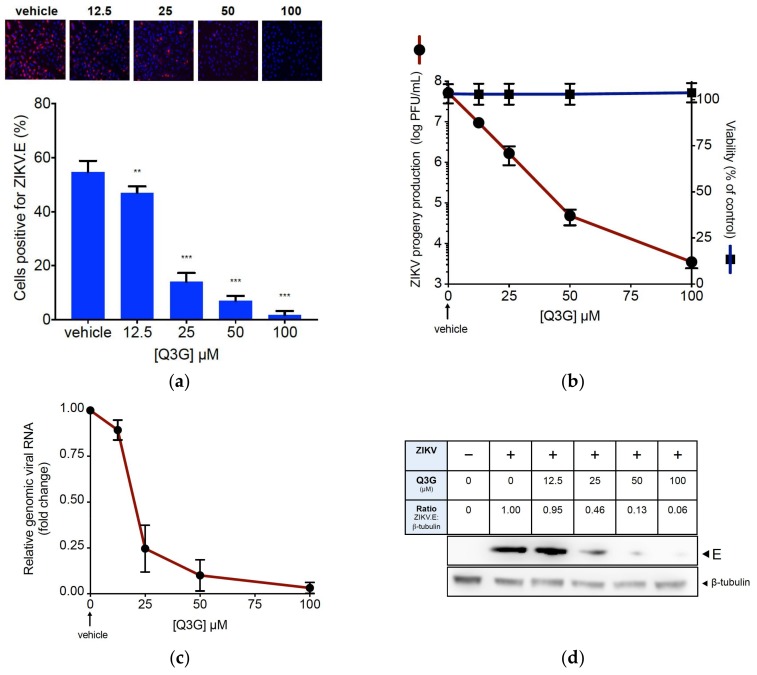
Q3G prevents infection of A549 cells by epidemic strain of ZIKV. A549 cells were infected 24 h with PF-25013-18 at a multiplicity of infection (MOI) of 2 in presence of increasing concentrations of Q3G or vehicle. In (**a**), immunofluorescence analysis of viral protein expression in ZIKV-infected A549 cells. The ZIKV envelope E protein (ZIKV.E) (red) and nuclei (blue) were visualised by fluorescence microscopy. The same magnification of ×20 was used throughout. Results from a representative experiment (*n* = 4 repeats) are shown. Bottom panels: quantification of positive cells for the viral protein E expression in ZIKV-infected A549 cells. In (**b**), ZIKV progeny production was quantified by plaque-forming assay (PFU). Data represent the means ± SD of four independent experiments performed in triplicate. In (**c**), the amount of viral genomic RNA in ZIKV-infected A549 cells was determined by RT-qPCR. Results are expressed as fold change of viral RNA transcripts in ZIKV-infected and Q3G-treated cells relative to those in vehicle-treated cells. Data represent the means ± SD of four independent experiments performed in triplicate. In (**d**), detection of intracellular E protein in ZIKV-infected A549 cells by immunoblot assay using anti-E mAb. β-tubulin served as loading control. Bands were quantified by densitometry using ImageJ software (Version 2.0.0; National Institutes of Health, Bethesda, MD, USA) and the ZIKV.E:β-tubulin ratio values are indicated in the table.

We reported that ZIKV PF-25013-18 clinical isolate from French Polynesia during the epidemic in 2013 replicates efficiently in human lung epithelial A549 cells [37]. First, we determined the Q3G cytotoxicity in A549 cells using a 3-[4,5-dimethylthiazol-2-yl]-2,5-diphenyltetrazolium bromide (MTT) assay (Figure S1). The highest non-cytotoxic concentration of Q3G was 200 µM. To assess whether Q3G inhibited contemporary epidemic ZIKV strain, A549 cells were infected 24 h with PF-25013-18 at the multiplicity of infection (MOI) of 2. ZIKV-infected A549 cells were incubated with increasing concentrations of Q3G up to 100 µM from the beginning of infectious virus cycle. By immunofluorescence (IF) analysis using anti-flavivirus E mAb 4G2, we showed that Q3G treatment resulted in a severe viral growth restriction in A549 cells (Figure 1a). At the non-cytotoxic concentration of 100 µM, Q3G reduced the viral progeny production by at least 4 log_10_ (Figure 1b) and the amount of intracellular viral RNA by 90% (Figure 1c). Also, viral protein production was severely restricted in a concentration-dependent manner (Figure 1d). We concluded that Q3G is a potent inhibitor of ZIKV growth in human cells.

**Figure 2:**
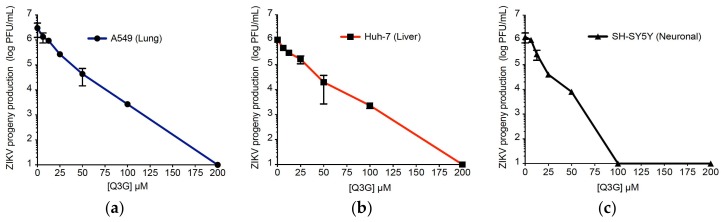
Q3G inhibits ZIKV growth across three human cell lines. Viral clone ZIKV MR766^MC^ was used to infect A549 (**a**) and Huh-7 (**b**) cells at MOI of 1 and SH-SY5Y (**c**) cells at MOI of 10. Increasing concentrations of Q3G or vehicle were added concurrently to virus input. Virus progeny production was determined at 48 h post-infection. Data represent the means ± SD of four independent experiments performed in triplicate.

We next investigated whether Q3G also protects human hepatoma Huh-7 and neuroblastoma SH-SY5Y cells from ZIKV infection. We recently reported that ZIKV MR766^MC^, viral clone derived from historical African strain MR766-NIID [38] replicates efficiently in A549 and SH-SY5Y cells [39]. As shown in Figure 2b, the growth of MR766^MC^ was also efficient in Huh-7 cells. First, we determined the sensitivity of Huh-7 and SH-SY5Y cells to increasing concentrations of Q3G using a MTT-based cell viability assay (Figure S1). Dose-dependent experiments showed that cell viability was reduced by Q3G at concentrations higher than 200 µM with a CC_50_ up to 600 µM (Table 1).

To evaluate the anti-ZIKV activity of Q3G in different human cell lines, A549, Huh-7, and SH-SY5Y were infected two days with MR766^MC^ at MOI of 1 or 10 in the presence of increasing concentrations of Q3G (Figure 2). At the highest non-cytotoxic concentration of Q3G, there was no viral progeny production regardless the human cell lines tested. At 100 µM, Q3G reduced by 3 log_10_ virus progeny production in A549 and Huh-7 cells (Figure 2a,b) whereas viral growth was still undetectable in SH-SY5Y cells (Figure 2c). The concentration that inhibited 90% of virus growth (IC_90_) was obtained using nonlinear regression, following the construction of a sigmoidal concentration–response curve. The IC_90_ was 15 µM (SH-SY5Y cells), 50 µM (Huh-7 cells), and 32 µM (A549 cells). Based on the determined cytotoxicity and antiviral efficacy, we calculated Q3G selectivity index (SI = CC_50_/IC_50_) which ranges from 23 to 60 (Table 1). These results confirmed that Q3G is a potent inhibitor of ZIKV infection across human cells.

### 2.2. Q3G Targets Early Stages of ZIKV Infection

**Figure 3:**
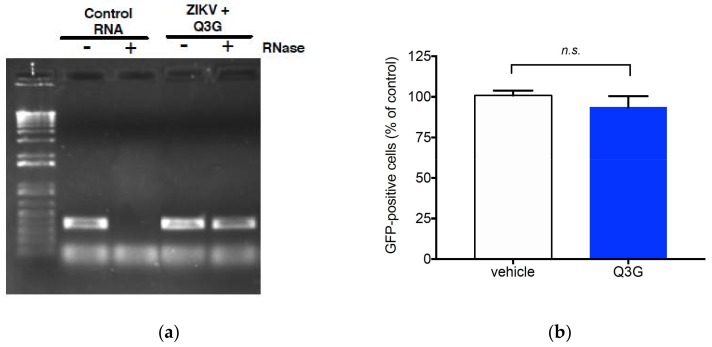
Effect of Q3G on ZIKV infectivity. In (**a**), RNase A protection assay on ZIKV. MR766^MC^ was incubated with 100 µM Q3G or mock-treated in presence of RNase A for 1 h at 37 °C. Viral RNA was extracted and amplified by RT-PCR with ZIKV.E primers. Viral RNA extracted from ZIKV and treated with RNase A or vehicle served as controls. Results from a representative experiment (*n* = 3 repeats) are shown. In (**b**), Viral inactivation assay. ZIKV_GFP_ was incubated with 100 µM Q3G or mock-treated at 37 °C for 1 h and the mixture was assessed for viral infectivity on A549 cells at MOI of 0.5 (2 µM Q3G final concentration). At 24 h p.i., the percentage of GFP-positive cells was determined by FACS analysis. Data represent the means ± SD of four independent experiments performed in triplicate.

To further investigate the mechanisms of Q3G-mediated inhibition of ZIKV, A549 cells were infected 24 h by the GFP-expressing mutant ZIKV_GFP_ derived from MR766^MC^ at the MOI of 0.5. We reported that ZIKV_GFP_ is a reliable tool to monitor viral growth [38]. At 50 µM of Q3G, the progeny production of ZIKV_GFP_ was reduced by 2 log_10_ and this was correlated with a reduction of GFP-positive A549 cells by 75% as determined by FACS analysis (Figure S2). RNase protection assay was first performed to determine whether Q3G induced the release of genomic RNA from extracellular virus particles. Viral RNA was insensitive to RNase suggesting that Q3G-mediated inhibition of ZIKV was not associated with a loss of viral particle integrity (Figure 3a). Furthermore, results from virus inactivation assays showed no reduction in virus infectivity when ZIKV_GFP_ was incubated with Q3G at 37 °C for 2 h (Figure 3b). These results indicated that Q3G does not cause disassembly of ZIKV particles nor affect their infectivity.

**Figure 4:**
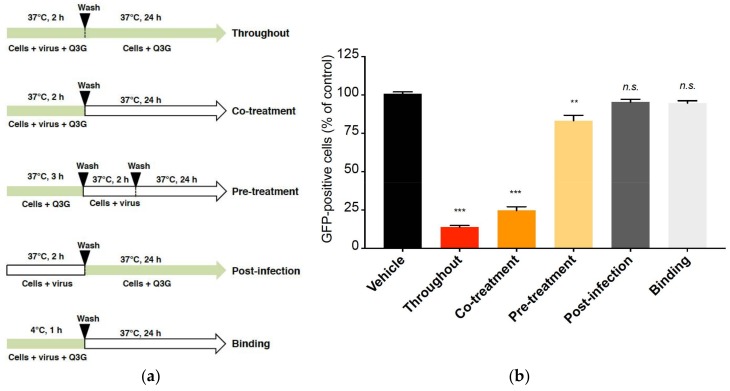
Q3G targets early stages of ZIKV infection. In (**a**), schematic representation of synchronised ZIKV_GFP_ infection and Q3G treatment assays in A549 cells. Q3G (100 µM) or vehicle were used for treatment of A549 cells throughout the infection (throughout), concurrently to virus input (co-treatment), pretreatment of naïve cells (pre-treatment), after virus exposure (post-infection) with specific washing steps and incubation periods. For viral attachment assay (binding), the test samples were used to treat A549 cells concurrently with ZIKV_GFP_ at 4 °C before washing and shifting the temperature to 37 °C. The parts of arrows colored in green or white indicate respectively the presence or the absence of Q3G during the infection. In (**b**), the percentages of GFP-positive cells were determined by flow cytometry assay. The data represent the means ± SD of four independent experiments performed in triplicate, and are expressed as relative values compared to vehicle-treated cells.

Next, we used the time-of-drug addition approach to determine which stages of ZIKV infection were targeted by Q3G [40]. As illustrated in Figure 4a, Q3G was added to A549 cells concurrently to virus input, prior to viral infection, or post-infection. As a positive control, Q3G was added throughout the infectious life cycle. We found that Q3G treatment concurrently with virus input severely reduced the percentage of GFP-positive A549 cells (Figure 4b, co-treatment), whereas little or no antiviral effect was observed when Q3G was added prior to infection or after virus exposure (Figure 4b, pre-treatment and post-infection). Such results suggested that Q3G essentially targets the initial stages of infectious life cycle rather than viral replication or viral assembly and release of virus particles. To determine whether Q3G precluded the attachment of virus particles to cell surface, pre-chilled ZIKV_GFP_ was mixed with Q3G and allowed to bind onto a A549 cell monolayer at 4 °C for 1 h followed by a temperature shift to 37 °C (Figure 4a, binding). The percentage of GFP-positive cells was determined 24 h after the temperature shift. There was no difference in GFP expression, suggesting that the incapacity of ZIKV to initiate productive infection in the presence of Q3G was not related to a defect in cell-attachment (Figure 4b, binding).

### 2.3. Q3G Inhibits ZIKV Internalisation in A549 Cells

**Figure 5:**
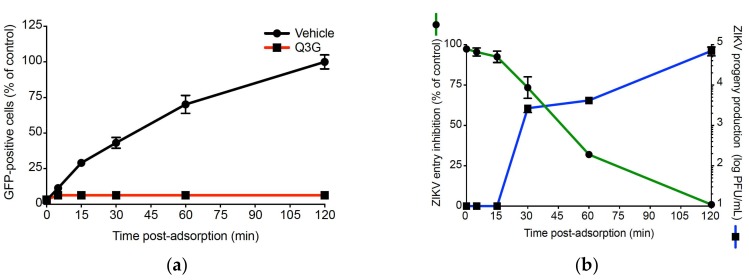
Q3G inhibits ZIKV entry in A549 cells. A549 cells were incubated 1 h with ZIKV_GFP_ at 4 °C and temperature was shifted to 37 °C in absence (vehicle) or presence of 100 µM Q3G. The percentage of GFP-positive cells was determined at 24 h p.i. In (**a**), attached and non-internalized virus particles were removed with citrate buffer at different time points post-adsorption. In (**b**), Q3G was added at different time points post temperature shift. The green curve represents the inhibition of ZIKV entry calculated from the percentage of GFP-positive cells. The blue curve represents the virus titers. Data represent the means ± SD of four independent experiments performed in triplicate.

Time-of-drug addition assays showed that Q3G-mediated inhibition of ZIKV relates to a post-adsorption step of the infectious virus cycle. To investigate whether Q3G inhibits internalisation of virus particles, A549 cells were incubated with ZIKV_GFP_ at 4 °C for 1 h to allow virus adsorption and temperature was then shifted to 37 °C to start virus penetration, in presence or not of Q3G (Figure 5a). At different time points post-adsorption, attached but not internalized virus particles were removed using citrate buffer. FACS analysis showed that, in the absence of Q3G, the percentage of GFP-positive A549 cells linearly increased to reach 50% at 45 min and a maximum at 120 min post temperature shift (Figure 5a). At 100 µM of Q3G, ZIKV_GFP_-infected A549 cells remained negative for GFP expression. These results suggest that Q3G is a fast and potent inhibitor of virus internalisation acting on virus particles trafficking early after the initial binding to plasma membrane. 

To evaluate how early Q3G is able to inhibit endocytosis of virus particles after plasma membrane binding, ZIKV_GFP_ was allowed to bind to the cell surface at 4 °C followed by a temperature shift to 37 °C. At different time points after temperature shift, cells were extensively washed and then incubated with Q3G. ZIKV_GFP_-infected A549 cells were examined for virus progeny production and GFP-expression at 24 h post-infection (Figure 5b). Until 15 min after temperature shift, Q3G treatment resulted in a complete absence of viral progeny, which was correlated with a reduction of GFP-positive cells by 90%. The inhibition of ZIKV infection was much less pronounced if Q3G was added 30 min after the temperature shift. Thus, the Q3G-mediated inhibition of ZIKV growth occurs early after virus binding to the plasma membrane and could be explained by the incapacity of plasma membrane-associated virus particles to be internalized into the host cell.

### 2.4. Quercetin, Hyperoside, and Kaempferol Are Inefficient against ZIKV

**Figure 6:**
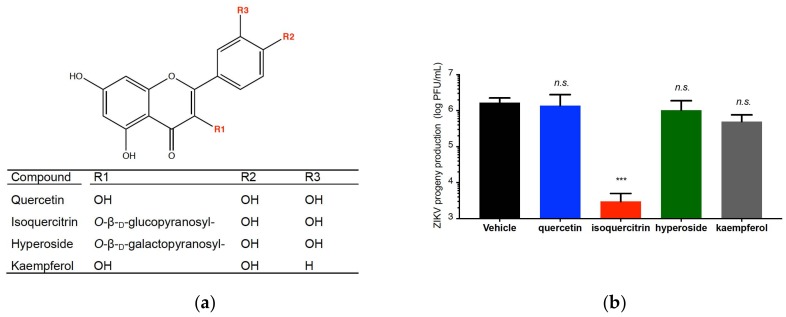
ZIKV sensitivity to the selected flavonoids. In (**a**), chemical structure of quercetin, isoquercitrin, hyperoside and kaempferol. In (**b**), A549 cells were infected with ZIKV MR766^MC^ in presence of 100 µM of each flavonoid and virus progeny production was determined at 24 h p.i. Data represent the means ± SD of four independent experiments performed in triplicate.

Then, we asked whether other flavonoids structurally related to Q3G, could exhibit anti-ZIKV properties. Three flavonoids were selected because of their close structural homologies with Q3G: quercetin, kaempferol, and hyperoside (Figure 6a). Their higher non-cytotoxic concentrations on A549 cells were determined using a standard MTT assay (Figure S3). ZIKV-infected A549 cells were incubated with either quercetin, hyperoside, or kaempferol and virus progeny production was analysed 24 h post-infection, Q3G was used as a positive control. At the higher non-cytotoxic concentrations, we found that quercetin, kaemfperol, and hyperoside showed no significant antiviral effect against ZIKV infection when compared to Q3G (Figure 6b). These results showed that the antiviral action of Q3G is not shared by other related flavonoids. Because quercetin was inefficient against ZIKV, we suggest that the sugar moiety might play an important role in Q3G capacity to inhibit virus entry in human cells.

### 2.5. Concluding Remarks

In the present study, our data demonstrated that flavonol glucoside isoquercitrin is a potent inhibitor of ZIKV across different cell types of human origin with a remarkable SI up to 60. Importantly, isoquercitrin is effective against ZIKV strains of African and Asian lineages. Whether or not isoquercitrin also exerts antiviral properties against other important mosquito-borne flaviviruses—such as dengue virus, yellow fever virus, West Nile virus, and Japanese encephalitis virus—which all are responsible for important communicable diseases worldwide is a critical issue that remains to be investigated. It is worth thinking that isoquercitrin could be a very attractive antiviral compound as its toxicity and pharmacokinetics are fairly well studied and its administration is well tolerated in humans [41].

As described for EGCG and curcumin, isoquercitrin precludes the initiation of ZIKV infection in the host cell. It has recently been reported that clathrin-mediated endocytosis pathway involving Axl/Gas6 as entry factors may play a key role in ZIKV entry into the host cell [9]. Whether Q3G-mediated inhibition of ZIKV entry involves Axl/Gas6 is also an open question that remains to be urgently answered. Interestingly, we showed that hyperoside bearing a galactose sugar moiety failed to inhibit virus infection. Such observation suggests that the type of sugar bound to the aglycone has an impact on the capacity of flavonoid to exert antiviral effect against ZIKV. To clarify the matter, it is necessary to develop structure–activity relationship (SAR) studies on the flavonoid. Such SAR studies would broaden our understanding of the mechanisms that contribute to flavonol glucoside antiviral action against medically-important pathogens such as ZIKV and other related mosquito-borne viruses, as well as influenza and Ebola viruses [24,42].

## 3. Materials and Methods

### 3.1. Cells, Virus, and Reagents

Human lung epithelial A549 cells (ATCC, CCL-185, Manassas, VA, USA), Vero cells (ATCC, CCL-81), human-derived Huh-7 hepatoma cells (ATCC, PTA-8561) and human neuroblastoma SH-SY5Y cells (ATCC, CRL2266) were grown in minimum essential medium (MEM: Gibco/Invitrogen, Carlsbad, CA, USA) supplemented with non-essential amino acids and 10% heat-inactivated fetal bovine serum (Dutscher, Brumath, France), under a 5% CO_2_ atmosphere at 37 °C. ZIKV strains PF-25013-18, MR766^MC^ and the mutant ZIKV_GFP_ have been previously described [38,43]. The ZIKV progeny production is determined by measuring the quantity of infectious virus particles released into the supernatant of infected cells by plaque-forming assay on Vero cells as previously described [38]. Q3G, hyperoside, kaempferol, and quercetin were purchased from Sigma-Aldrich (Saint-Quentin-Fallavier, France) and stock solutions were prepared in sterile dimethyl sulfoxide (DMSO, Sigma-Aldrich). Growth culture medium supplemented with 0.2% of DMSO was used as a vehicle control. The mouse anti-pan flavivirus envelope E protein mAB 4G2 was purchased from RD Biotech (Besançon, France).

### 3.2. Cytotoxicity Assay

The cytotoxicity was evaluated by spectrophotometric MTT (3-[4,5-dimethylthiazol-2-yl]-2,5-diphenyltetrazolium bromide) assay as described previously [37]. The concentration that inhibited viability in 50% of cells (CC_50_) was obtained by performing nonlinear regression following the construction of a sigmoidal concentration–response curve (variable slope; Graphad Prism; La Jolla, CA, USA).

### 3.3. Immunofluorescence and Flow Cytometry Assays

For immunofluorescence assay, cells grown on glass coverslips were fixed with 3.7% formaldehyde at room temperature for 10 min. Fixed cells were permeabilized with Triton X-100 (0.15%) in PBS for 4 min and stained using the mouse anti-pan flavivirus envelope E protein mAb 4G2 (1:1000 dilution). Nucleus was stained with DAPI (4′,6-diamidino-2-phenylindole). The coverslips were mounted with Vectashield (Vector Labs, Premanon, France), and fluorescence was observed using a Nikon Eclipse E2000-U microscope (Nikon, Lisses, France). Images were captured and treated using a Hamamatsu ORCA-ER camera (Hamamatsu, Japan) and the imaging software NIS-Element AR (Nikon). For flow cytometry assay, cells were fixed with 3.7% paraformaldehyde in PBS for 20 min, washed twice with PBS, and then submitted to a flow cytometric analysis using FACScan flow cytometer (BD Bioscience, Le Pont-de-Claix, France). Results were analysed using FlowJo software (BD Bioscience).

### 3.4. Virus Binding and Internalisation Assays

For binding assay, A549 cell monolayers were pre-chilled at 4 °C for 30 min and subsequently infected with ZIKV in presence or absence of Q3G for 60 min at 4 °C. After infection, cells were washed twice with ice-cold PBS to remove unbound virus [44]. The cells were further incubated with fresh medium at 37 °C for 24 h before being subjected to cytometry assay. For internalisation assay, A549 cells monolayers were pre-chilled at 4 °C for 30 min and subsequently infected with ZIKV for 1 h at 4 °C. Cells were washed with PBS then treated with citrate buffer (pH 3, citric acid 40 mM, potassium chloride 10 mM, sodium chloride 135 m) for 1 min to remove attached but non-internalized virus [44]. Cells were then washed with PBS and further incubated with fresh medium for 24 h at 37 °C before being subjected to cytometry and virus titration assays.

### 3.5. RNase Protection Assay

RNase protection assay was performed as previously described with some minor modifications [45]. Briefly cell-free virus particles were incubated with Q3G and 15 µg·mL^−1^ RNase A (USB-Affymetrix, Sigma-Aldrich) for 1 h at 37 °C, and viral RNA was extracted using a QIAamp Viral RNA Mini Kit (Qiagen, Courtaboeuf, France). The viral RNA samples were subjected to RT-PCR and gel electrophoresis. The RNA was reverse transcribed using 50 pmol of random hexamers (Eurofins, Nantes, France) and MMLV reverse transcriptase (Promega, Charbonnières-les-bains, France). PCR amplification was performed using GoTaq polymerase (Promega) with ZIKV.E primers (forward 5′-GTCTTGGAACATGGAGG-3′ and reverse 5′-TTCACCTTGTGTTGGGC-3′), which were designed to match both MR766-NIID and PF-25013-18 sequences.

### 3.6. Viral Inactivation Assay

Viral inactivation assay was performed as previously described with minor modifications [46]. Briefly, ZIKV_GFP_ particles (6.7 log PFU) was mixed with 100 µM Q3G and then incubated at 37 °C for 1 h. As a control, a same dose of ZIKV_GFP_ was mixed with Q3G and then directly tested without an incubation period. Prior addition of the sample on A549 cells grown on a six-well plate, the virus-Q3G mix was diluted to 50-fold in growth cell medium in order to reduce Q3G concentration below its effective dose against ZIKV and to get a virus input about 1 PFU/cell. After 2 h of adsorption at 37 °C, the samples were discarded and the cells were washed twice with PBS. The cells were further incubated with fresh medium at 37 °C for 24 h before being subjected to cytometry as above described.

### 3.7. RT-qPCR

Total RNA including genomic viral RNA was extracted from cells with RNeasy kit (Qiagen) and reverse transcribed using 500 ng of total RNA, as above described. Quantitative PCR was performed on an ABI7500 Real-Time PCR System (Applied Biosystems, Life Technologies, Villebon-sur-Yvette, France). Briefly, 10 ng of cDNA were amplified using 0.2 μM of each primer and 1× GoTaq Master Mix (Promega). Data were normalised to the internal standard *GAPDH*. For each single-well amplification reaction, a threshold cycle (Ct) was calculated using the ABI7500 program (Applied Biosystems, Life Technologies) in the exponential phase of amplification. Relative changes in gene expression were determined using the ΔΔCt method and reported relative to the control. The couple of primers for ZIKV.E gene have been described elsewhere [37].

### 3.8. Western Blot Analysis

Cells were lysed in RIPA buffer and cell lysates were analysed by immunoblot assay as previously described [47]. Primary antibodies were prepared at 1:1000 dilutions. Secondary antibodies:anti-rabbit immunoglobulin-horseradish peroxidase and anti-mouse immunoglobulin-horseradish peroxidase conjugates were prepared at 1:2000 dilutions. Blots were revealed with ECL detection reagents.

### 3.9. Data Analysis

Statistical analysis consisted of one-way ANOVA followed by Dunnett’s test for multiple comparisons with a significance of *p* < 0.05. All statistical tests were performed using Prism software (version 7.0; GraphPad software, La Jola, CA, USA). Degrees of significance are indicated as follows: * *p* < 0.05; ** *p* < 0.01; *** *p* < 0.001, n.s. = not significant.

## Figures and Tables

**Table 1:** Cytotoxicity and anti-ZIKV activity of Q3G.

Human Cell Lines	CC_50_ (µM) ^a^	IC_50_ (µM) ^b^	IC_90_ (µM) ^c^	SI ^d^
A549	551.2 ± 43.2	15.5 ± 2.3	32.0 ± 3.4	35.6
Huh-7	326.8 ± 45.7	14.0 ± 3.8	50.0 ± 4.7	23.3
SH-SY5Y	582.2 ± 41.4	9.7 ± 1.2	15.0 ± 2.3	60.0

Cytotoxic concentration (CC) and inhibitory concentration (IC) were obtained by performing nonlinear regression followed by the construction of a sigmoidal concentration–response curves from Figure 2 and Figure S1. ^a^ Concentration that inhibited cell viability by 50%; ^b,c^ concentrations that inhibited MR766^MC^ progeny production by 50 or 90%; ^d^ Selectivity index (CC_50_/IC_50_).

## Supplementary Materials

Supplementary materials can be found at http://www.mdpi.com/1422-0067/19/4/1093/s1.

## Abbreviations

A549	Human lung epithelial cell line
DAPI	4′,6-diamidino-2-phenylindole
ECL	Enhanced chemiluminescent substrate
EGCG	Epigallocatechin gallate
FACS	Fluorescence-activated cell sorting
GAPDH	Glyceraldehyde-3-phosphate dehydrogenase
GFP	Green fluorescent protein
HCV	Hepatitis C virus
Huh-7	Human hepatoma cell line
IF	Immunofluorescence
mAB	Monoclonal antibody
MOI	Multiplicity of infection
p.i.	post infection
PBS	Phosphate-buffered saline
PFU	Plaque forming unit
Q3G	Quercetin-3-*O*-glucoside or isoquercitrin
RIPA	Radio immune precipitation assay
SH-SY5Y	Human neuroblastoma cell line
ZIKV	Zika virus
ΔΔCt	Cycle threshold
