# Supplementary material for: The Flavonoid Isoquercitrin Precludes Initiation of Zika Virus Infection in Human Cells
Source: Int J Mol Sci. 2018 Apr 5;19(4):1093. doi: 10.3390/ijms19041093 (PMC5979602; doi:10.3390/ijms19041093)
Supplement: Supplementary file 1 [file ijms-19-01093-s001.pdf]

The Flavonoid Isoquercitrin Precludes Initiation of Zika Virus Infection in Human Cells

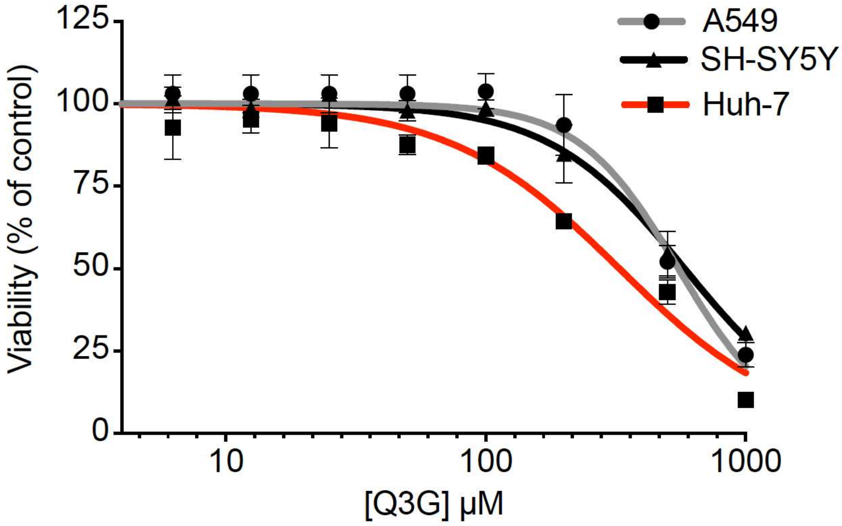

Figure S1. Three human cell lines were evaluated for their sensitivity to increasing concentrations of Q3G using a MTT assay. The cytotoxic test was performed 72 h post-treatment. Data represent the means ± SD of four independent experiments performed in triplicate.

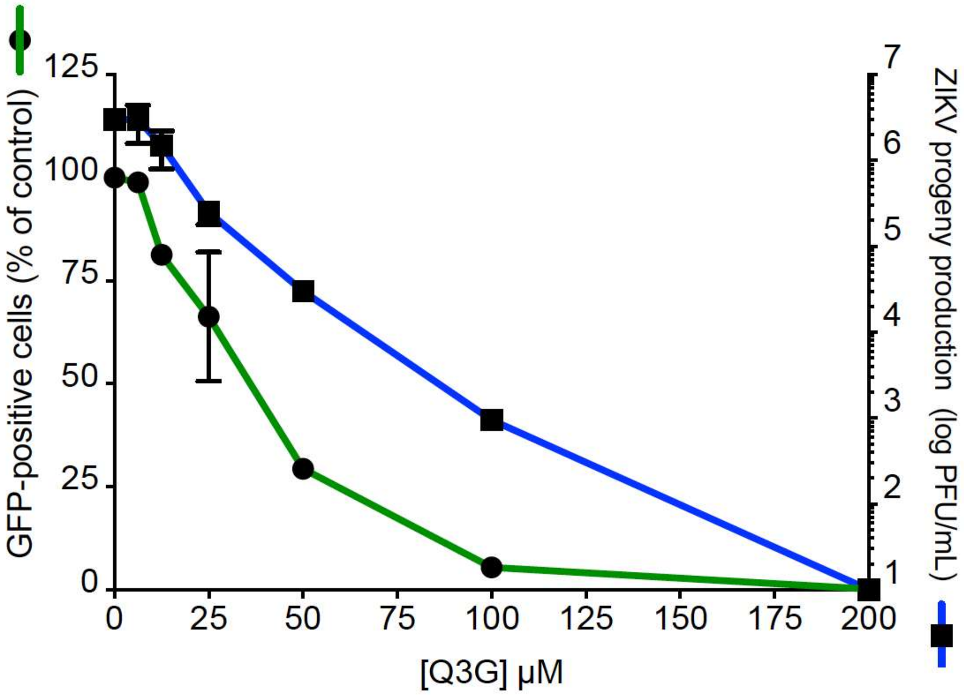

Figure S2. Antiviral effect of Q3G on mutant ZIKV expressing GFP. A549 cells were infected with $ZIKV_{GFP}$ in presence of increasing concentrations of Q3G. At 24 h p.i., the percentage of GFP-expressing cells was determined by FACS analysis (left axis) and virus progeny production was assessed (right axis). The data represent the means ± SD of four independent experiments performed in triplicate and are expressed as relative values compared to vehicle.

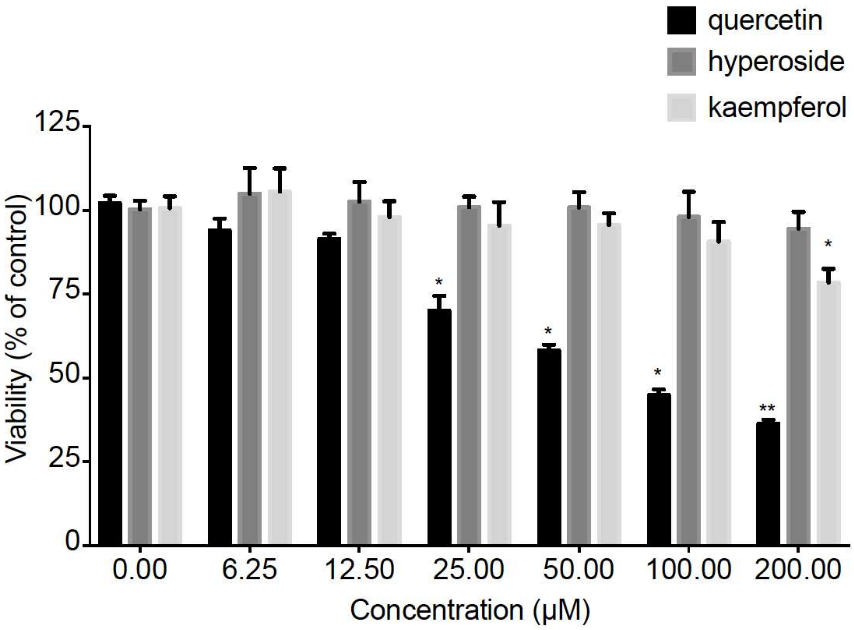

Figure S3. Cytotoxicity of selected flavonoids. Different concentrations of flavonoids were used to treat A549 cells to determine cytotoxicity using MTT assay. The cytotoxic test was performed 72 h post-treatment. Data represent the means ± SD of four independent experiments performed in triplicate.
